# Knowledge diffusion of dynamical network in terms of interaction frequency

**DOI:** 10.1038/s41598-017-11057-8

**Published:** 2017-09-07

**Authors:** Jian-Guo Liu, Qing Zhou, Qiang Guo, Zhen-Hua Yang, Fei Xie, Jing-Ti Han

**Affiliations:** 1 Data Science and Cloud Service Research Centre, Shanghai University of Finance and Economics, Shanghai, 200433 P.R. China; 2 Research Center of Complex Systems Science, University of Shanghai for Science and Technology, Shanghai, 200093 P.R. China; 3 School of Finance, Shanghai University of Finance and Economics, Shanghai, 200433 P.R. China; 4 Business School, Huzhou University, Huzhou, 313000 P.R. China

## Abstract

In this paper, we present a knowledge diffusion (SKD) model for dynamic networks by taking into account the interaction frequency which always used to measure the social closeness. A set of agents, which are initially interconnected to form a random network, either exchange knowledge with their neighbors or move toward a new location through an edge-rewiring procedure. The activity of knowledge exchange between agents is determined by a knowledge transfer rule that the target node would preferentially select one neighbor node to transfer knowledge with probability *p* according to their interaction frequency instead of the knowledge distance, otherwise, the target node would build a new link with its second-order neighbor preferentially or select one node in the system randomly with probability 1 − *p*. The simulation results show that, comparing with the Null model defined by the random selection mechanism and the traditional knowledge diffusion (TKD) model driven by knowledge distance, the knowledge would spread more fast based on SKD driven by interaction frequency. In particular, the network structure of SKD would evolve as an assortative one, which is a fundamental feature of social networks. This work would be helpful for deeply understanding the coevolution of the knowledge diffusion and network structure.

## Introduction

With the rapid growth of network science^1, 2^, the studies on knowledge diffusion in social networks have attracted much research attention^3, 4^. Regarding knowledge diffusion in social networks, two research issues come to the fore, i.e. how knowledge is diffused in a social network^5–7^, and how the social network itself, in which knowledge is diffused, evolves^8–10^. Inspired by these endeavors, a recent focal research direction in the field of complex networks is coevolutionary dynamics, which combines the topological evolution of the network with the knowledge dynamics in the network nodes^11–13^. Various research efforts have been reported, e.g. Bosch *et al*.^14^ investigated how knowledge environments coevolve with the emergence of organization forms and combinative capabilities, Cowan and Jonard^15^ analyzed the relationship between network architecture and the performance of knowledge diffusion, and found that the steady-state level of average knowledge is maximal when the network structure is the small-world, Palazzolo *et al*.^16^ analyzed on the coevolution of the social and knowledge networks by using the agent based model, Roth and Cointet^17^ argued that the dynamics of the communities can be described as the coevolution of a socio-semantic network. More recently, Liu *et al*.^18^ argued that the knowledge diffusion processes and the structure evolution were always evolving simutaneously. Havakhor and Soror^19^ focused on the reputation mechanisms and the distribution of knowledge roles to enhance and enable the smooth transfer of knowledge in the social media networks. Li *et al*.^20^ argued that cooperative learning network (CLN) was a uniform network with small-world characteristics and leader nodes played important role in the knowledge spreading process on CLN.

The previous contributions on the coevolutionary adaptive networks show a promising research direction. Furthermore, the factors affecting the knowledge diffusion of dynamical network include knowledge distance^21–23^, reputation mechanisms^24–26^, interaction frequency^27–29^, etc^30, 31^. Firstly, based on the knowledge transfer rule, which showed that the knowledge transfer was most effective when the knowledge distance was neither too large nor too small^32, 33^, Luo *et al*.^34^ explored the possible dynamic patterns of network evolution and knowledge diffusion driven by the knowledge distance and found that the bi-directional influences between knowledge transfer and neighborhood adjustment gave rise to the coevolution of the network structure and the diffusion of knowledge at the global level. Secondly, reputation mechanisms, as a specific characteristics of the social media networks (SMNs) would enhance and enable, respectively, the smooth transfer of knowledge in a SMN. Wang *et al*.^35^ proposed the collaboration reputation for trustworthy Web service selection in social networks. In addition, another factor affecting the knowledge diffusion is interaction frequency because individuals who communicate with each other frequently are more likely to share knowledge than those who communicate infrequently^6^. Pentland^36^ argued that interaction frequency could affect the performance of knowledge diffusion. Hudson *et al*.^37^ indicated that social media use was positively related with brand relationship quality. Peng *et al*.^38^ described the complexity and uncertainty of social influence by friend entropy and interaction frequency entropy. Moreover, interaction frequency is the key factor that affects the career development of agents^39, 40^, which can be regard as the evolution of social network. Therefore, we argue that the interaction frequency is an important factor affecting the performance of knowledge transfer.

**Figure 1:**
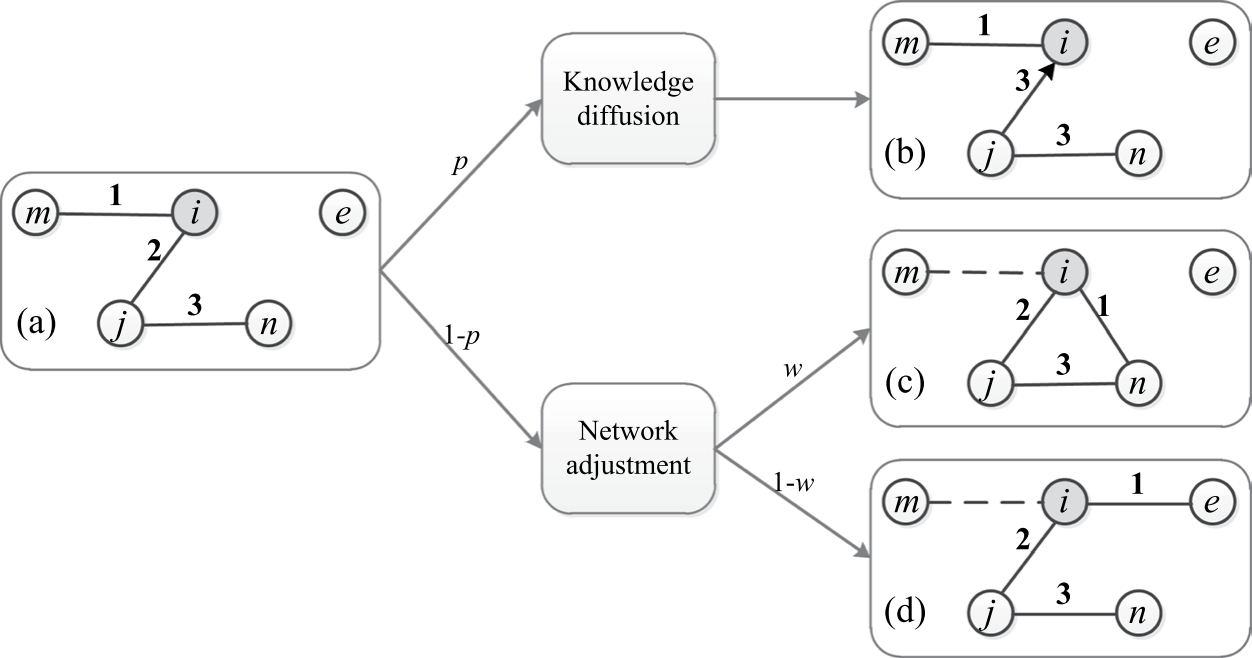
Schematic illustration of the presented model. The nodes represent the agents and the edge weight *τ*_*i,j*_ represents the interaction frequency between the agents. The grey node *i* represents the target node which is randomly selected, and the node *i* will exchange knowledge with one of the neighbors or build new link with other nodes. Subplot (a) shows the original network structure. As the sender, node *j* and *m* are the neighbors of node *i*. The node *n* is one of the neighbors of the node *j*, while the node *e* is another randomly selected node. And subplot (b) shows the knowledge diffusion process of node *i* exchanging knowledge with node *j*. The network structure evolution is shown in the subplot (c) and (d), while the dash line is the broken edge.

Inspired by the above ideas, by taking into account the influence of interaction frequency, we present a social knowledge diffusion model, namely SKD model, to explore the coevolutionary of knowledge diffusion and dynamical networks. At each time step (see Fig. 1), with probability *p*, a target node would select a candicate to exchange knowledge in its neighbor node set according to their interaction frequency. And with probability 1 − *p*, the node would rebuild its relationship with a new node in its second-order neighbors or randomly select one node in the system. The experimental results show that, comparing with the Null model defined by the random selection mechanism and the traditional knowledge diffusion (TKD) model driven by the knowledge distance, the knowledge diffusion speed of the SKD model is faster than the knowledge diffusion speed of the Null and TKD model. More importantly, the network structure would finally evolves as assortative which is one key factor of social networks, which suggests the interaction frequency plays an important role in the knowledge diffusion of the organizations.

## The Social Knowledge Diffusion (SKD) Model

For the social knowledge diffusion (SKD) model, suppose there is a random network with *N* nodes and *E* links. Each node *i* has an initial knowledge level *ν*_*i*_(0). And the interaction frequency *τ*_*i,j*_(0) is set to 1 if node *i* and *j* are connected; Otherwise, *τ*_*i,j*_(0) = 0. The selection process is defined by Eq. (1). The neighbor *j* of the node *i* will be selected to diffuse knowledge at time *t* with probability *P*_*i,j*_(*t*) given by:

1 $${P}_{i,j}(t)=\frac{{\tau }_{i,j}(t)}{\sum _{i\in {{\rm{\Gamma }}}_{i}(t)}{\tau }_{i,j}(t)},$$

where the Γ_*i*_(*t*) shows the neighbor sets of node *i* at time *t*, the *τ*_*i,j*_(*t*) denote the interaction frequency between node *i* and *j* at time *t*. At each timestep, if there is an interaction between node *i* and *j*, the interaction frequency *τ*_*i,j*_(*t*) would be:

2 $${\tau }_{i,j}(t)={\tau }_{i,j}(t-\mathrm{1)}+1.$$

The overall procedure of the proposed SKD model is comprised of the following steps.

**Step 1:** The initial network is set as a random network with *N* nodes and *E* links, and initialize each node’s knowledge level.

**Step 2:** Randomly select a node from the sets of node as the target node *i*;

**Step 3:** The target node could either exchanges knowledge with a neighbor node or rebuild a new link with its second-order neighbor preferentially or select one node in the system randomly.

- With probability *p*, one neighbor *j* of the target node *i* will be selected for knowledge transfer with probability *P*_*i,j*_(*t*) by Eq. (1). Then, calculating the new value for node *i*′*s* knowledge:
  3 $${v}_{i}(t+1)={\nu }_{i}(t)+{\alpha }_{i,j}(t)\max \{{\nu }_{j}(t)-{\nu }_{i}(t),\,0\}$$
  where ${\alpha }_{i,j}(t)\in \mathrm{(0},\mathrm{1)}$ is absorptive capability of target node *i*. The higher interaction frequency, the more ease of knowledge transfer^41^. Therefore, we assume that
  4 $${\alpha }_{i,j}(t)=\frac{1}{1+{e}^{\tfrac{1}{{\tau }_{i,j}(t)}}}$$
- With probability 1 − *p*, the target node *i* rebuild its connections. Procedurally, the network adjustment process is accomplished by the following steps:
  With probability *w*, node *i* rewire to one neighbor of its original neighbors, the target original neighbor will be selected with according to Eq. (1); at the same time, the neighbor of the target original neighbor will be selected to be rewired according to Eq. (1). Otherwise (i.e. with probability 1 − *w*) to a random one selected from the whole population except for its nearest neighbors
  If node *i* has been successfully rewired to a new neighbor at step (i), one of its original links is to be removed. Remove the link between node *i* and its one neighbor which has the smallest interaction frequency.

**Step 4**: Repeat the step 2 and step 3 until the count of the iterations reaches the pre-specified upper-limit *T*_*max*_.

## The Null Model

In order to explore the effects of interaction frequency in the coevolution of knowledge diffusion and network structure, a Null model is introduced in the following way:

**Step 1:** The initial network is set as a random network with *N* nodes and *E* links, and initialize each node’s knowledge level.

**Step 2:** Randomly select an node as the target node *i*;

**Step 3:** The target node either exchanges knowledge with a neighbor node or rebuild its connections defined by random selection mechanism.

**Step 4:** Repeat the step 1 and step 2 until the count of the iterations reaches the pre-specified upper-limit *T*_*max*_.

## The Traditional Knowledge Diffusion (TKD) Model

For the traditional knowledge diffusion (SKD) model, suppose there is a random network with *N* nodes and *E* links. Each node *i* has an initial knowledge level ν_*i*_(0). And the interaction frequency *τ*_*i,j*_(0) is set to 1 if node *i* and *j* are connected; Otherwise, *τ*_*i,j*_(0) = 0. The overall procedure of the proposed TKD model is comprised of the following steps.

**Step 1:** The initial network is set as a random network with *N* nodes and *E* links, and initialize each node’s knowledge level.

**Step 2:** Randomly select a node from the sets of node as the target node *i*;

**Step 3:** The target node could either exchanges knowledge with a neighbor node or rebuild a new link with its second-order neighbor preferentially or select one node in the system randomly.

- With probability *p*, the node agent learns new knowledge from one of the neighbors using knowledge transfer (KT) rule, which is based on the measurement of the interacting nodes’ knowledge distance. When the knowledge distance is larger than a given threshold we predict no gain from their interaction as the lack of common knowledge hinders learning. Setting the threshold value of “knowledge distance” as *d* and calling it as “knowledge-exchange threshold”. Then, calculating the gain for node i from the interaction with node *j*: First, calculate the knowledge distance between node i and node j:
  5 $${d}_{ij}=\sum _{t}{\rm{\max }}\{{\nu }_{j,t}-{\nu }_{i,t},\,0\}$$
  Then, calculate the actual gain:
  6 $$G=\{\begin{array}{c}min\{max\{{\nu }_{j,t}-{\nu }_{i,t},0\},s\},{d}_{ij} < d\\ \mathrm{0,}\,{d}_{ij}\ge d\end{array}$$
  where *d*_*ij*_ is less than the threshold *d*, node *i* obtains a limited amount of knowledge which in no larger than a given upper-bound *s* from node j. When the knowledge distance is less than *s*, the larger the distance is, the more node *i* gains. Otherwise, when *d*_*ij*_ exceeds *d*, there is no gain from the interaction. After the interaction and knowledge exchange, the new value for node i’s knowledge is calculated as:
  7 $${\nu }_{i}(t+\mathrm{1)}={\nu }_{i}(t)+G$$
- With probability 1 − *p*, a network adjustment or NA rule is applied so that the focal node rewires one existing link to a new node.

**Step 4:** Update the knowledge status quo of each node and the entire network structure.

**Step 5:** Repeat steps (2), (3), and (4) until the count of the iterations reaches the pre-specified upper-limit T max.

## Measurements

In this paper, we use the average knowledge stock $\bar{V}(t)$ of the entire population at time *t* to measure the knowledge diffusion performance, and use the assortative coefficient *r*(*t*) to measure the property of network structure.

The average knowledge stock $\bar{V}(t)$ is defined as

8 $$\bar{V}(t)=\frac{1}{N}\sum _{i=1}^{N}{\nu }_{i}(t)$$

which measures how efficient the system could diffuse the knowledge.

The assortative coefficient *r*(*t*) is one important feature for networks, which is defined as

9 $$r(t)=\frac{1}{{\sigma }_{q}^{2}(t)}\sum _{jk}jk({\varepsilon }_{j,k}(t)-{q}_{j}(t){q}_{k}(t)),$$

where *q*_*k*_(*t*) is the degree distribution representing the probability that the degree of a randomly selected neighbour of one node is *k* at time *t*. The ${\varepsilon }_{j,k}(t)$ is the joint probability distribution of the remaining degrees. The ${\sigma }_{q}^{2}(t)$ represents the variance of the remaining degrees distribution *q*_*k*_(*t*). The assortative coefficient *r*(*t*) ranges from −1 to 1.

## Results

In order to measure the performance of the knowledge diffuses in the SKD and Null model, we investigate the average knowledge stock $\bar{V}(t)$ and the assortative coefficient *r*(*t*) for two models.

**Figure 2:**
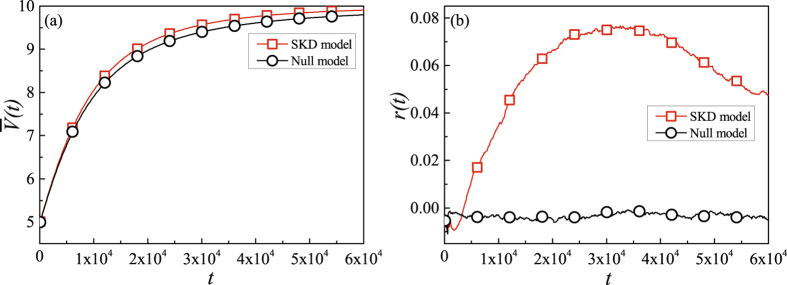
The results of knowledge diffusion and network structure evolution in SKD model and Null model (*w* = 0.3 and *p* = 0.5). Each simulation result is obtained by averaging over 100 independent runs.

Figure 2(a) shows the average knowledge stock $\bar{V}(t)$ of the SKD and Null model. The result shows that, the average knowledge stock $\bar{V}(t)$ of the system rapidly increases in early periods of the evolution, with the increasing speed gradually declines. As the time goes, one could find that the average knowledge stock growth in the SKD model is quicker than that in the Null model, which indicates that defining the knowledge absorptive capability with interaction frequency will exaggerate the efficiency of knowledge diffusion. Figure 2(b) shows the assortative coefficient *r*(*t*) as a function of time step *t*. One can find that the assortative mixing *r* decreases at first and then increases, at the very early stage ($t\in \mathrm{[0,}\,\mathrm{4000]}$) of the simulations. Then, the assortative mixing *r*(*t*) increases to 0.07 in the SKD model while the assortative mixing *r*(*t*) of the Null model are close to 0. Comparing with the Null model leading to disassortative, one can find that the network structure would evolve as assortative networks.

**Figure 3:**
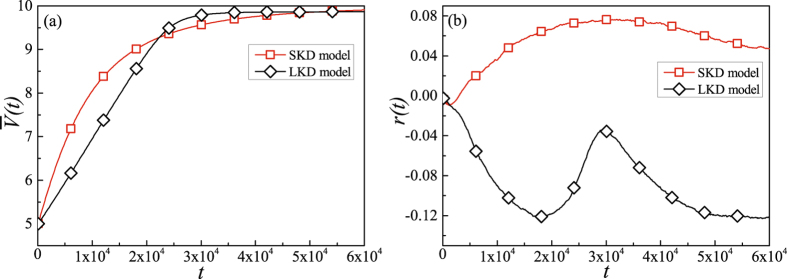
The results of knowledge diffusion and network structure evolution in the SKD model and TKD model (*w* = 0.3 and *p* = 0.5). Each simulation result is obtained by averaging over 100 independent runs. When the time step *t* = 30000, in the TKD model, the average knowledge stock $\bar{V}(t)$ reach a stable state of the average knowledge stock $\bar{V}(t)=9.7$ and the assortative coefficient $r(t)=-0.035$ while $r(t)=0.76$ in SKD model.

Furthermore, we compare the performance of SKD model with the performance of traditional knowledge diffusion (TKD) model^34^ in which the exchange candidate is selected in terms of the knowledge distance. Figure 3(a) shows that the knowledge would spread faster in the SKD model before the time step *t* = 22000, resulting in the average knowledge stock $\bar{V}(t)=9.3$. Meanwhile, Fig. 3(b) shows that the evolution of network structure tends to be assortative while the one of the TKD model tends to be disassortative.

**Figure 4:**
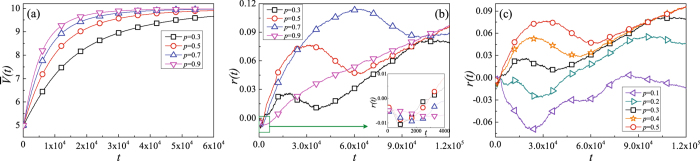
The influence of the change of *p* on knowledge diffusion and network structure(*w* = 0.3). Each point is obtained by averaging over 100 independent runs.

The effect of the parameter *p* on knowledge diffusion can be evaluated more thoroughly by comparing the time-variances of the average knowledge stock $\bar{V}(t)$ and assortative coefficient *r*(*t*) on the different values of *p*. The result is shown in Fig. 4.

From Fig. 4(a), one can find that the average knowledge stock $\bar{V}(t)$ of the system rapidly increases at early stage, possibly due to the knowledge disequilibrium between the nodes. As the knowledge transfer process evolves, nodes barely receive knowledge from their neighbors, resulting in a stable state of the average knowledge stock $\overline{V}(t)=9.7$. Figure 4(b),(c) shows the assortative coefficient *r*(*t*) as a function of the time step *t* with the different parameter *p*. From the subgraph of Fig. 4(b), one can find that the assortative coefficient decreases at first and then increases, at the very early stage ($t\in \mathrm{[0,}\,\mathrm{4000]}$). But once the knowledge transfer has been process evolves, the assortative coefficient *r*(*t*) turn to decrease. Figure 4(b) shows that, when the timesteps *t* < 25000, the assortative coefficient *r*(*t*) tend to increase slower as the *p* increases while $0.5\le p\le 0.9$. This may result from that as *p* becomes smaller the network structure changes more frequently. Besides the previous phenomena, Fig. 4(c) shows that, the assortative coefficient *r*(*t*) is smaller for a smaller *p* than that for a larger *p* while 0.1 ≤ *p* ≤ 0.5. And when *p* ≤ 0.2, the network structure may evolve as disassortative.

In the proposed model, $w\in \mathrm{(0},\mathrm{1)}$ is a parameter that measures the global mobility of the nodes. The node tends to rewire to second-order neighbor for great *w*, while the nodes tend to migrate toward remote locations for small *w*.

**Figure 5:**
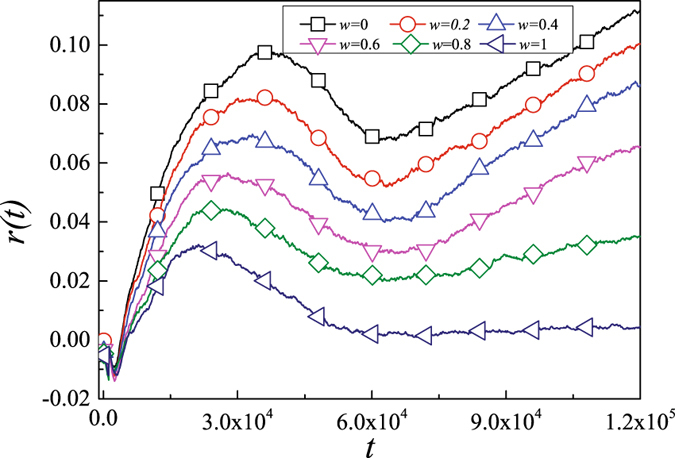
The influence of the change of *w* on knowledge diffusion and network structure(*p* = 0.5). Each simulation result is obtained by averaging over 100 independent runs.

Figure 5 shows the impact of *w* on the evolution of network structure. At the very early stage ($t\in \mathrm{[0,}\,\mathrm{4000]}$) of the simulations for all parameter *w*, one can find similar phenomenon is as that in the subplot of Fig. 4(b). When assortative coefficient *r*(*t*) increases, the assortative coefficient *r*(*t*) increases faster and uses more time to reach the top value as the decreasing of *w*. However, once the network structure adaptation has been processed long enough (t = 32000), the assortative coefficient *r*(*t*) turns to decrease. In more than about *t* = 70000, when the value of *w* = 1, the assortative coefficient *r*(*t*) will reach a steady state as time evolve while *w* < 1 the assortative coefficient *r*(*t*) tends to increase.

## Conclusion and Discussions

The knowledge diffusion in the networks often comes with the network structure evolution. In this paper, we present a social knowledge diffusion model (SKD) model for dynamic networks where the interaction frequency is taken into account for the knowledge diffusion process and the network structure evolution. In the knowledge diffusion process, with probability *p*, the target node would preferentially select one neighbor node to transfer knowledge who has the higher interaction frequency with the target node. Moreover, the higher interaction frequency is, the more knowledge will be transferred. In the network structure evolution, with probability 1 − *p*, the target node would build a new link with its second-order neighbor preferentially or randomly select one node in system, and break the link with one neighbor node who has the smallest interaction frequency with the target node.

To measure the performance of the SKD model, we implement two groups of comparative experiments on the Null model defined by the random selection mechanism and the traditional knowledge diffusion (TKD) model driven by the knowledge distance. We apply the average knowledge stock $\bar{V}(t)$ to evaluate the diffusion efficiency, and use the assortative coefficient *r*(*t*) to measure the property of network structure. The assortative coefficient *r*(*t*) is one important feature of the network network structure^42, 43^. The simulation results show that the knowledge would spread more fast comparing with the Null model and TKD model. In particular, comparing with the TKD model leeding to disassortative, the network structure evolves as assortative networks which is an important feature of social network, while the assortative coefficient *r*(*t*) of the Null model are close to 0.

In addition, we also analyze the effect of the parameter *p* on the average knowledge stock $\bar{V}(t)$ and assortative coefficient *r*(*t*). The simulation results indicate that the average knowledge stock $\bar{V}(t)$ will become lager as the parameter *p* increases. But the lager *p* is not always beneficial to the network structure evolves as assortative. Further more, we investigate the effect of the parameter *w* on the assortative coefficient *r*(*t*). The parameter *w* is used to measures the global mobility of the nodes. The node tends to rewire to second-order neighbor for great *w*, while the nodes tend to migrate toward remote locations for small *w*. From the simulation results, one can find that the smaller parameter *w* the lager assortative coefficient *r*(*t*) will be.

The present work is limited in the following ways. Firstly, in our model, the network structure evolution has been taken into account. However, the initial network structure plays an important role in knowledge diffusion process^16^. Therefore, as another direction of the future work, we need to investigate. Secondly, in this paper, the knowledge has been represented by a stock. At the same time, how to measure the knowledge stock of a paper in the real life is an open question. Lastly, when applying the empirical data of the real social network evolution to statistically analyse the process of knowledge diffusion, we must address this problem. In the future work, it is a worthwhile problem to make empirical analysis on the knowledge diffusion process in the social network evolution.
